# Clinical Impact of ^18^F-FDG PET/CT on the Management of Gynecologic Cancers: One Center Experience

**DOI:** 10.22038/AOJNMB.2018.11208

**Published:** 2019

**Authors:** Akram Al-Ibraheem, Abedallatif AlSharif, Ramiz Abu-Hijlih, Imad Jaradat, Asem Mansour

**Affiliations:** 1 Department of Nuclear Medicine, King Hussein Cancer Center, Amman, Jordan; 2 Department of Radiology & Nuclear Medicine, Jordan University hospital, Amman, Jordan; 3 Department of Radiation Oncology, King Hussein Cancer Center, Amman, Jordan; 4 Department of Radiology, King Hussein Cancer Center, Amman, Jordan

**Keywords:** Gynecological malignancies, Negative predictive value, PET/CT

## Abstract

**Objective(s):**

We aim to investigate the clinical impact of ^18^F-FDG PET/CT in managing patients with gynecological malignancies and pelvic or extrapelvic lymph nodes that are of equivocal significance on conventional imaging.

**Methods:**

We retrospectively evaluated ^18^F-FDG PET/CT scans of patients with gynecologic tumors who were referred to King Hussein Cancer Center from January 2010 to August 2014. PET/CT results were compared with MRI and CT findings. We evaluated sensitivity and specificity of ^18^F-FDG PET/CT, its role in changing treatment plan and its positive predictive value (PPV) and negative predictive value (NPV).

**Results:**

Ninety seven patients (mean age: 49 years) underwent ^18^F-FDG/PET in the study period (40 cervical, 37 endometrial and 20 ovarian cancers). PET/CT scan provided additional information in 23 patients; upstaging 4.1% (4 patients; 3 true positive) and down staging in 19.5% (19 patients; 15 true negative). As a result, treatment strategy was changed from curative to palliative in three patients, and modification of radiation field or additional curative therapy was implemented following exclusion of distant metastasis in 11 patients. Mean follow up time for the whole cohort was 35 months (range 6 - 60 months). NPV of ^18^F-FDG PET/CT in detecting extrapelvic lymphadenopathy was 83.3%.

**Conclusion:**

^18^F-FDG PET/CT has high clinical impact in management of gynaecological cancers as it alters the treatment plan in a substantial number of patients who had equivocal findings on conventional imaging, as well as it offers excellent validity in lymph nodes staging.

## Introduction

The detection rate of gynecologic malignancies is increasing every year; mortality associated with gynecological cancers is high mainly due to detection of disease at advanced stage. Endometrial cancer is the most common malignancy of the female reproductive system followed by cervical cancer (1). Ovarian cancer ranks as the fifth highest cause of cancer related death among women and in some countries with higher prevalence and mortality in developing countries (2). Endometrial cancer is the 5th most common cancer in women after breast, colorectal, thyroid and lymphoma and is the most common gynecological malignancy affecting Jordanian females. There were 121 (4.5% of female cancers) new endometrial cancer cases diagnosed in 2013 according to the National Cancer Registry.  Ovarian Cancer is the seventh most common cancer affecting females in Jordan, there were 87 (23.3% of female cancers) cases in the year 2013, in addition there were 50 new cases of cervical cancer in 2013 (1.5% of female cancers) according to national Cancer Registry (3). 

Staging of gynaecological malignancies is generally performed according to the International Federation of Gynecology and Obstetrics system (FIGO) (4, 5). Although FIGO is principally based on clinical and surgical findings, it supports the use of radiological imaging for assessment of pelvic and extra pelvic lymph node status as well as detection of distant metastasis (4, 5). In fact, recent advances achieved in Computed Tomography (CT) and Magnetic Resonance Imaging (MRI) have enhanced the role of oncological imaging whether at initial staging or at assessment of treatment response. The revised FIGO staging system (2009) recommends the integration of cross-sectional imaging such as CT or MRI in assessing and planning treatment for cervical cancers (6). Both techniques however rely on anatomical data and morphological alterations in normal tissue appearance to characterize and diagnose malignancy (7), principally lymph nodes measuring > 1 cm in short axial diameter are considered pathological. Reliance on lymph node size, however and is challenged by frequent presence of enlarged non-diseased lymph nodes. Radiologic evaluation of residual tumor in known lesions becomes even more challenging, this gains particular importance in the setting of distorted post-surgical anatomy (8, 9).

There is a growing body of evidence supporting the role of ^18^F-Flourodeoxyglucose (^18^F-FDG) Positron Emission Tomography/Computed Tomography (PET/CT) in management of gynecologic malignancies (9-13). ^18^F-FDG PET is a functional imaging technique that portrays tumor metabolic activity regardless of lesion size or its related anatomical alterations. In addition to its established high sensitivity and specificity in detecting distant metastasis, ^18^F-FDG PET/CT is a reliable tool for preoperative lymph node staging with a high negative predictive value (NPV) (10).

Although ^18^F-FDG PET/CT has been integrated into the treatment algorithm of gynecological malignancies by many radiation oncologists, its clinical value still requires further evaluation (11-13). The impact of ^18^F-FDG PET/CT images for assessment of gynecologic malignancies requires further evaluation particularly for assessing indeterminate pelvic and para-aortic lymph nodes (11-13). Furthermore, clinical outcomes in patients with equivocal CT or MRI findings and negative ^18^F-FDG PET/CT scan have not been adequately evaluated. Conversely, ^18^F-FDG PET/CT positive lesions including detection of pathological para-aortic lymph nodes are included in Gross Tumor Volume (GTV) delineation, which is critical in determining radiation field design and boosting radiation dose (11-13).

In this one center study we aim to evaluate the clinical impact of ^18^F-FDG PET/CT in patients with gynecologic cancers and equivocal findings on conventional radiological imaging. This clinical scenario can present at staging or on follow up imaging, disease management may be modified or even changed based on extent of lymph node involvement. At KHCC ^18^F-FDG PET/CT is frequently used to solve this clinical problem. The value of ^18^F-FDG PET/CT in evaluating pelvic and extra pelvic lymph nodes has been demonstrated with histopathology after pelvic lymphadenectomy, its value however in confirming or excluding disease in patients with equivocal radiological findings particularly without histopathology record, was not adequately discussed in literature. At KHCC we design our management plan in cases with equivocal radiological findings according to ^18^F FDG PET/CT findings, the clinical outcome of these patients particularly when ^18^F FDG PET/CT is negative has not been adequately explored. 

## Methods

***Study population***

We conducted a retrospective study of the clinical, radiologic, radiotherapy, and histopathological records of patients with ovarian, endometrial and cervical cancer who were referred for ^18^F-FDG PET/CT at King Hussein Cancer Center (KHCC) during the period between January 2010 and August 2014.

At KHCC, patients with gynecological cancers routinely undergo complete routine staging workup including medical history taking, physical examination, tumor biopsy, cytology, cystoscopy, proctoscopy, colposcopy, full body CT scan and pelvic MRI at initial diagnosis. Multidisciplinary teams discuss imaging findings on patient by patient basis and ^18^F-FDG PET/CT is requested for patients with primary neoplasms which are generally known to be ^18^F-FDG avid with equivocal radiological findings. ^18^F-FDG PET/CT examination is also requested for patients with locally advanced disease. 

Routine follow up imaging in patients with gynecological malignancies includes chest, abdomen and pelvis CT scans and pelvic MRIs at three monthly intervals.^18^F-FDG PET/CT is also requested during post-treatment follow up to evaluate treatment response and to assess for disease relapse or distant metastasis. 

***Scanning protocol***

Contrast-enhanced CT scans of chest, abdomen and pelvis images were obtained using a 64-slice Brilliance Philips scanner set at 120 kV with auto mAs. Images were reconstructed using a 512×512 matrix at 3 mm thickness with 1.5 mm increments. 

Pelvic MRI scans were performed using 3.0 Tesla Ingenia Philips scanner. Turbo spin echo T1 and T2 weighted sequences were obtained prior to contrast injection in the axial and sagittal planes, diffusion-weighted imaging (DWI) was performed at b-values of 0, 500 and 800. 

^18^F-FDG PET/CT scans were performed using a hybrid PET/CT scanner (Gemini, Philips, USA). Subjects were required to be fasted for at least 6 hours prior to scanning. Fasting blood glucose levels were checked prior to radiotracer injection and scans were not be performed if the glucose levels were more than 200 mg/dL. Standard ^18^F-FDG PET/CT imaging protocol in gynecologic malignancies at KHCC starts 60 minutes after injection of 370 MBq ^18^F-FDG intravenously. Patients are requested to void their urinary bladder prior to performing a whole body scan. The PET scans are acquired in a caudal to cranial direction and CT scans are performed for attenuation correction and anatomical localization. No oral or intravenous contrast agents are administered thereafter. CT images are obtained in 5-millimeter thick sections at 80 mA and 140 kVp. PET images are subsequently reconstructed, corrected for attenuation and fused with CT images. 

***Image interpretation***

Two experienced nuclear medicine physicians assessed the images by consensus. The fused ^18^F- FDG PET/CT images are assessed for areas of high FDG uptake. Correlation was made with findings from conventional imaging such as CT scans and MRIs of the patients. Suspicious or equivocal lymph nodes that were detected by conventional imaging were re-assessed on ^18^F- FDG PET/CT. Lymph nodes with short axis more than 1.0 cm were considered pathological according to CT interpretation criteria. Lymph nodes that have FDG uptake higher than background uptake were considered pathological.

We assessed ^18^F-FDG PET/CT scans at initial staging as well as follow up re-staging periods for all our subjects. We staged the tumors according to (FIGO) staging guidelines. Reported imaging findings are defined according to the following criteria; scan is rated true positive (TP) if it provides accurate positive staging/restaging, true negative (TN) if it provides accurate negative staging/restaging, false positive (FP) if it provides wrong positive result and false negative (FN) if it provides wrong negative results according to clinical outcome.

***Statistical analysis***

Descriptive statistics and frequency distributions were generated for all the clinical data. 

Data are presented as means and standard deviations (SDs) or frequencies and percentages. Data was analyzed statistically using SPSS-15 version. Sensitivity, specificity, Positive Predictive Value (PPV) and Negative Predictive Value (NPV) of ^18^F-FDG PET/CT scans in assessing presence of gynaecological malignancies were calculated based on histopathological examination findings, clinical outcome and results of serial follow up conventional imaging scans considered as the gold standard.

## Results

A total of 97 patients with gynecologic cancers were included in this study. Table 1 summarizes patients’ age, histopathology and indication of ^18^F-FDG PET/CT. Overall,^ 18^F-FDG PET/CT findings were concordant with CT and MRI in 74 patients (76.3 %). On the other hand,^ 18^F-FDG PET/CT provided additional information in 23 patients (23.7%).

There were 23 patients with equivocal radiological findings on CT and or MRI. ^18^F-FDG PET/CT upstaged 4 patients and down staged 19 patients, detailed description of the role of ^18^F-FDG PET/CT in upstaging and down staging these patients is seen in Table 2. All the four patients who had their condition upstaged had locally advanced cervical disease; one patient had distant metastasis in abdominal lymph nodes, one patient had abdominal and mediastinal lymph nodes, on patient had sternal bone metastasis. The fourth case was a 36 year-old lady, who was initially treated for localized cervical cancer; however a restaging ^18^F-FDG PET/CT revealed local recurrence and metastasis to supraclavicular lymph nodes. Biopsies were obtained from the cervix and supraclavicular lymph nodes; histopathology of the cervix revealed local recurrence, while histopathology of excised lymph nodes did not reveal any malignant cells. Therefore, this was a false positive case of the supraclavicular lymph nodes metastases which was encountered in both ^18^F-FDG PET/CT and conventional CT scans. 

Interestingly, 19 patients were down-staged by ^18^F-FDG PET/CT that involved 7 pelvic and 12 extra-pelvic tumor sites. Follow-up of the down-staged patients revealed four false negative cases. In all the four false negative cases, PET/CT failed to detect borderline enlarged pelvic or retroperitoneal lymph nodes or peritoneal soft tissue deposits. On the other hand, in the remaining patients (15 patients; true negative ^18^F-FDG PET/CT) did not reveal any evidence of disease at PET negative sites (11 with pelvic and/or extra-pelvic lymph nodes and 4 with suspected visceral metastasis) during follow up. Furthermore, pulmonary metastases were excluded in 3 patients and hepatic metastasis was excluded in 1 patient (mean follow up 21 months). According to ^18^F-FDG PET/CT findings treatment plan was modified in a total of 14 patients (Table 3).

The sensitivity, specificity, PPV and NPV of initial ^18^F-FDG PET/CT staging of gynaecological malignancies were 96%, 100%, 100% and 90%, respectively. The sensitivity, specificity, PPV and NPV of ^18^F-FDG PET/CT PET restaging were 93.9%, 80%, 95.8% and 72.7%, respectively (Table 4). NPV of ^18^F-FDG PET/CT in detecting extrapelvic lymphadenopathy was 83.3%.

## Discussion

FIGO staging system relies mainly on clinical findings including physical examination, cystoscopy, proctoscopy, colposcopy, and biopsy results (4, 5). Diagnostic imaging, though not endorsed by FIGO, is increasingly used to assess lymph node status and look for distant metastases or evidence of local disease extent. Although lymphadenopathy is not included in the FIGO staging system, it is considered a major factor in selecting treatment plan at initial staging because nodal involvement is considered the best prognostic indicator in patients with gynecologic malignancies (14).^ 18^F-FDG PET/CT has been recently endorsed by the National Comprehensive Cancer Network (NCCN) practice guidelines for cervical and endometrial cancer workup (15, 16). Nevertheless, the lack of consensus guidelines on pre-treatment abdominopelvic imaging for endometrial cancer reflects the diversity in practice among institutions worldwide (10-14). At KHCC the role of ^18^F-FDG PET/CT is recognized as a powerful tool in oncologic imaging, it is currently employed as the mainstay of diagnostic imaging in several cancers such as lung cancers and lymphomas. The utilization of ^18^F-FDG PET/CT in gynecologic malignancies is being increasingly applied and its value in clinical practice at KHCC is widely accepted among our clinicians 

Our patients with ^18^F-FDG-avid metastatic lesions other than para-aortic lymph node are shifted to palliative care, whereas patients with localized disease will be offered radical treatment. In addition, we modify radiation plans by assigning areas that are reported negative on ^18^F-FDG PET to a low dose radiotherapy region; these areas are sometimes entirely excluded from radiation field.

**Table 1:** Patients’ characteristics and indication for ^18^F-FDG PET/CT scan

**Site of primary neoplasm**	**Cervical cancer**	**Endometrial cancer**	**Ovarian Cancer**
Number of patients	40	37	20
Histopathology	40 Squamous cell carcinoma	37 Adenocarcinoma	14 epithelial tumors 4 Germ cell tumor 2 clear cell carcinomas
Mean Age (years)	44 (Range: 28-65)	54 (range: 43-80)	51 (40-68)
PET done at Staging	17	9	12
PET done at Re-staging	23	26	8

**Table 2:** Contribution of ^18^F-FDG PET/CT to patient staging in different gynecological cancers patients with equivocal radiological findings

**Primary malignancy**		**Cervical cancer**	**Endometrial cancer**	**Ovarian cancer**
Contribution of patient to changing patient stage	No change	24	34	16
	Up-staging	4 (1 False positive)	0	0
	Down-staging	12 (1 false negative)	3 (1 false negative)	4 (2 false negative)
Method of Final diagnosis	Follow-up Imaging	13	2	2
	Histopathology	3	1	2
Mean follow up (months)		37.7(Range: 8-60)	32.1(Range:6-58)	34.5 (Range: 9-56)

**Table 3:** Contribution of ^18^F-FDG PET/CT to change in patient management

		**Cervical cancer**	**Endometrial cancer**	**Ovarian Cancer**
**PET changing management**	Modify Radiation Plans	2	2	0
	Palliative to Curative Intent	6	1	0
	Curative to palliative Intent	3	0	0

**Table 4:** Sensitivity, Specificity, PPV and NPV of ^18^F-FDG PET/CT for all patients, at staging and restaging, respectively

	**Staging and Restaging**	**95% CI**	**Staging**	**95% CI**	**Restaging**	**95% CI**
**Sentivivity**	94.9%	87.39% to 98.59%	96.6%	82.24% to 99.91%	93.9%	83.13% to 98.72%
**Specificity**	89.5%	66.86% to 98.70%	100.0%	66.37% to 100.00%	80.0%	44.39% to 97.48%
**PPV**	97.4%	90.88% to 99.28%	100.0%	100% to 100%	95.8%	86.92% to 98.76%
**NPV**	81.0%	61.77% to 91.79%	90.0%	56.74% to 98.41%	72.7%	46.04% to 89.29%

**Figure 1:**
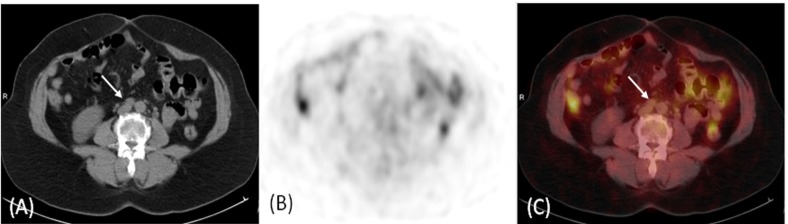
A 55 year-old female patient with cervical cancer underwent ^18^F -FDG PET/CT for staging. (A) Transaxial CT scan images demonstrated suspicious aorto-caval lymph nodes (arrow). Corresponding transaxial ^18^F -FDG PET (B) and transaxial fused ^18^F -FDG PET/CT (C) images didn’t demonstrate increase in FDG uptake in these lymph nodes (arrow). Patient received radical treatment to the pelvic diseases and follow up didn’t demonstrate any progression in these retroperitoneal lymph nodes

**Figure 2:**
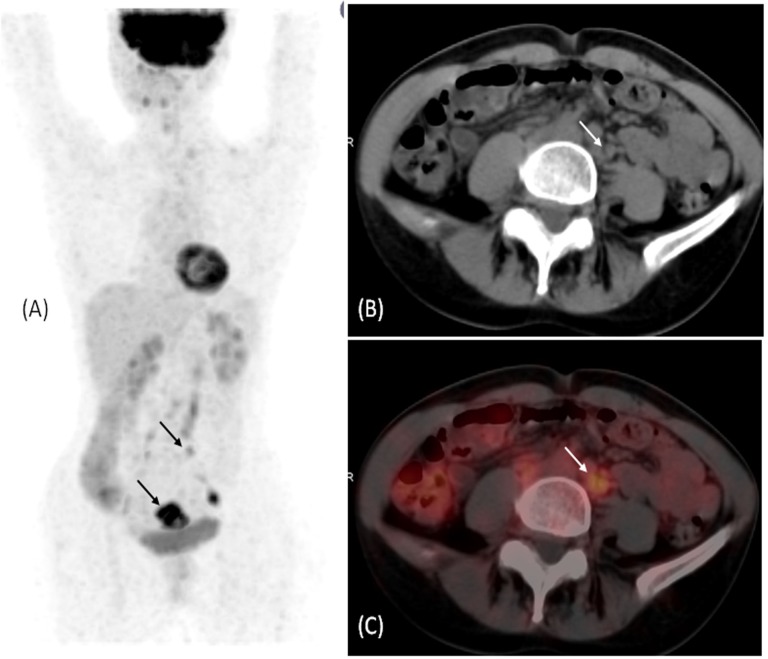
A 60 year-old female patient with cervical cancer underwent ^18^F-FDG PET/CT for staging. (A) Maximum intensity projection (MIP) of the ^18^F-FDG PET demonstrated the hypermetabolic cervical tumor in the pelvis (arrow) with multiple foci of abnormal increased FDG uptake in the pelvis and abdomen (arrow). (B) Transaxial CT scan demonstrated subcentimetric retroperitoneal lymph nodes (arrow) not categorized as pathological based on morphologic criteria. (C) Transaxial fused ^18^F-FDG PET/CT image in the abdomen demonstrated multiple hypermetabolic retroperitoneal lymph nodes (arrow) correlating to the subcentimetric lymph nodes seen on CT scan and the focal abnormal increased FDG uptake seen on MIP

Patients with negative^ 18^F-FDG PET/CT despite radiological suspicious extra-pelvic lymph nodes are considered negative for metastasis; therefore, radiotherapy field is limited to the pelvic region in these patients. A positive ^18^F-FDG PET/CT finding in extra-pelvic lymph nodes implies extending the treatment plan to include the diseased nodes. No histopathological confirmation of ^18^F-FDG avid lymph nodes is performed in our standard protocol at KHCC, this actually resulted in the high specificity and high PPV of our results particularly in staging PET/CT scans, where a positive pelvic lymph node was considered metastatic and the patient was treated accordingly. However, biopsy was performed in selected cases that had positive findings detected on ^18^F-FDG PET/CT imaging of extra-pelvic lymph nodes.

In a retrospective study of 47 early-stage cervical cancers, the accuracy and NPV of ^18^F-FDG PET/CT in assessing lymph node status was 99.5% and 99.3%, respectively, irrespective of lymph node size(14). Our results also compare well with several other studies that have confirmed the impact of ^18^F-FDG PET/CT in staging patients with early stage or clinically-suspected ovarian cancer. In a recent prospective study, ^18^F-FDG PET/CT demonstrated high accuracy in detecting retroperitoneal lymphadenopathy (87% accuracy) in patients with suspected ovarian cancer (17). Signorelli et al. (2013) had studied the role of ^18^F-FDG PET/CT in detecting nodal metastases in confirmed early ovarian cancer cases and detected sensitivity, specificity, accuracy, PPV and NPV of 75.5%, 99.4%, 98.1%, 87.5% and 98.6%, respectively, by site-based analysis based on histopathology findings (18). 

The high NPV of ^18^F-FDG PET/CT in predicting lymph node status was also demonstrated in patients with endometrial cancer with an NPV of 97.2% on a nodal site basis and 93.1% on a patient basis (18). These staging studies were performed using histopathology of surgically excised lymph nodes after pelvic and retroperitoneal lymphadenectomy as gold standard to calculate sensitivity, specificity and diagnostic accuracy of ^18^F-FDG PET/CT in patients with gynecological malignancies. Several authors, however; have demonstrated increased morbidity in patients who received combined surgery and radiotherapy (19). Furthermore, Cochrane review and NCI consensus found no evidence that pretreatment surgical paraaortic lymph node assessment for locally advanced cervical cancer is beneficial; on the contrary surgical approach could potentially have an adverse effect on survival (20). In addition, the impact of lymphadenectomy in patients with ovarian or endometrial is extremely controversial (21, 22). Our findings, therefore confirm the value of ^18^F-FDG PET/CT in a clinical setting where management of disease can be safely based on ^18^F-FDG PET/CT findings.

Moreover, there is growing body of evidence supporting the use of ^18^F-FDG PET/CT in the diagnostic work-up of patients with gynecologic malignancies. Therefore, many radiation oncologists currently incorporate ^18^F-FDG PET/CT in external-beam radiotherapy planning by modifying the treatment field (9-13). ^18^F-FDG PET/CT-based radiotherapy optimization allows improved tumor-volume dose distribution and detailed 3D-dosimetric evaluation of risk organs while minimizing treatment-related toxicity and avoiding increased surgical morbidity due to staging lymphadnectomy (9-13). Our retrospective results have demonstrated comparable efficacy of ^18^F-FDG PET/CT versus reported efficacy of surgical laparatomy and therefore is expected to reduce surgically induced morbidity and help avoid unnecessary surgery. This particular clinical scenario is faced frequently in clinical practice and is not adequately explored in literature. At King Hussein Cancer Center (KHCC) in Amman, Jordan we integrate ^18^F-FDG PET/CT findings in the process of defining treatment intent and refining radiotherapy plans of high risk patients, patients with locally-advanced disease and in patients with equivocal lymph node status or suspicious distant metastatic disease on conventional workup.

To the best of our knowledge, the clinical implication of applying ^18^F-FDG PET/CT scan in routine clinical practice, which does not include pelvic and retroperitoneal lymphadenectomy, has not been adequately evaluated (13). It is currently established that the incorporation of ^18^F-FDG PET/CT avid lesions in the radiation field results in better disease control (23), on the other hand, the clinical outcome of patients with negative ^18^F-FDG PET/CT results and suspicious CT or MRI findings who were assigned to a low dose regimen is not known. Our study yielded high NPV of ^18^F-FDG PET/CT particularly at staging in this understudied subset of patients. 

In this paper we present our experience using ^18^F-FDG PET/CT in guiding treatment decisions of gynecological malignancies. Overall, our results show that management plans were changed in 14 out of 23 patients who had discrepant findings between PET/CT and conventional imaging (at staging or restaging). The presence of distant FDG-avid lesions switched management plans to a palliative approach in 3 patients. Furthermore, 11 patients with equivocal and suspicious extra-pelvic radiologic findings proved to have pelvic localized disease on ^18^F-FDG PET/CT and were therefore offered therapeutic radical radiotherapy and modified radiation fields (Figure 1).

^18^F-FDG PET/CT positive extra-pelvic lesions were included in Gross Tumor Volume (GTV) delineation (Figure 2). This approach is considered critical to determine radiation field design and boost dose as shown in our previous literature review (13). What is more interesting than defining radiotherapy field extent on staging ^18^F-FDG PET/CT in the current analysis, is the high NPV of ^18^F-FDG PET/CT. PET/CT was able to conclusively exclude hepatic and lung metastasis (mean follow up 21 months) and exclude extra pelvic metastatic lymphadenopathy (mean follow up 27 months); with excellent clinical outcome. 

The limitation of our study is the diverge range of gynaecological malignancies. We were also not able to include the evaluation of disease-free or overall survival rates since our study involved a heterogeneous group of patients with different primary malignancies at variable FIGO stages. In addition, histopathologic confirmation of pelvic lymph node status was also not obtained in patients with ^18^F-FDG avid pelvic lymph nodes. 

## Conclusion

^18^F-FDG PET/CT has a high clinical impact in management of patients with gynecological tumors. ^18^F-FDG PET/CT can guide patient management as it can modify treatment plan in a substantial number of patients as well as it offers excellent validity in lymph nodes staging. The high NPV of ^18^F-FDG PET/CT in patients with equivocal radiological findings allows confident exclusion of nodal metastases. 

## References

[1] Perroy A, Kotz H (2010). Cervical cancer. The Bethesda handbook of clinical oncology.

[2] (2016). Key statistics about ovarian cancer.

[3] Nimiri O (2012). Cancer incidence in Jordan. Non-Communicable Diseases Directorate, Jordan Cancer Registry.

[4] FIGO Committee on Gynecologic Oncology (2014). FIGO staging for carcinoma of the vulva, cervix, and corpus uteri. Int J Gynaecol Obstet.

[5] Prat J; FIGO Committee on Gynecologic Oncology (2014). Staging classification for cancer of the ovary, fallopian tube, and peritoneum. Int J Gynaecol Obstet.

[6] Pecorelli S, Zigliani L, Odicino F (2009). Revised FIGO staging for carcinoma of the cervix. Int J Gynaecol Obstet.

[7] Suppiah S, Kamal SH, Mohd Zabid AZ, Abu Hassan H (2017). Characterization of adnexal masses using multidetector contrast-enhanced CT scan–recognising common pitfalls that masquerade as ovarian cancer. Pertanika J Sci Technol.

[8] Hricak H, Gatsonis C, Chi DS, Amendola MA, Brandt K, Schwartz LH (2005). Role of imaging in pretreatment evaluation of early invasive cervical cancer: results of the intergroup study American College of Radiology Imaging Network 6651–Gynecologic Oncology Group 183. J Clin Oncol.

[9] Magné N, Chargari C, Vicenzi L, Gillion N, Messai T, Magné J (2008). New trends in the evaluation and treatment of cervix cancer: the role of FDG–PET. Cancer Treat Rev.

[10] Musto A, Grassetto G, Marzola MC, Chondrogiannis S, Maffione AM, Rampin L (2014). Role of 18F-FDG PET/CT in the carcinoma of the uterus: a review of literature. Yonsei Med J.

[11] Rockall AG, Cross S, Flanagan S, Moore E, Avril N (2012). The role of FDG-PET/CT in gynaecological cancers. Cancer Imaging.

[12] Cihoric N, Tapia C, Krüger K, Aebersold DM, Klaeser B, Lössl K (2014). IMRT with 18 FDG-PET\CT based simultaneous integrated boost for treatment of nodal positive cervical cancer. Radiat Oncol.

[13] Salem A, Salem AF, Al-Ibraheem A, Lataifeh I, Almousa A, Jaradat I (2011). Evidence for the use PET for radiation therapy planning in patients with cervical cancer: a systematic review. Hematol Oncol Stem Cell Ther.

[14] Sironi S, Buda A, Picchio M, Perego P, Moreni R, Pellegrino A (2006). Lymph node metastasis in patients with clinical early-stage cervical cancer: detection with integrated FDG PET/CT 1. Radiology.

[15] Koh WJ, Greer BE, Abu-Rustum NR, Apte SM, Campos SM, Chan J (2013). Cervical cancer. J Natl Compr Canc Netw.

[16] Koh WJ, Greer BE, Abu-Rustum NR, Apte SM, Campos SM, Chan J (2014). Uterine neoplasms version 12014. J Natl Compr Canc Netw.

[17] Michielsen K, Vergote I, Op de Beeck K, Amant F, Leunen K, Moerman P (2014). Whole-body MRI with diffusion-weighted sequence for staging of patients with suspected ovarian cancer: a clinical feasibility study in comparison to CT and FDG-PET/CT. Eur Radiol.

[18] Signorelli M, Guerra L, Pirovano C, Crivellaro C, Fruscio R, Buda A (2013). Detection of nodal metastases by 18F-FDG PET/CT in apparent early stage ovarian cancer: a prospective study. Gynecol Oncol.

[19] Landoni F, Maneo A, Colombo A, Placa F, Milani R, Perego P (1997). Randomised study of radical surgery versus radiotherapy for stage Ib-IIa cervical cancer. Lancet.

[20] Brockbank E, Kokka F, Bryant A, Pomel C, Reynolds K (2013). Pretreatment surgical para-aortic lymph node assessment in locally advanced cervical cancer. Cochrane Database Syst Rev.

[21] Kim HS, Ju W, Jee BC, Kim YB, Park NH, Song YS (2010). Systemic lymphadnectomy for survival in epithelial ovarian cancer: a meta-analysis. Int J Gynecol Cancer.

[22] Wright JD, Huang Y, Burke WM, Tegas AI, Hou JY, Hu JC (2016). Influence of lymphadenectomy on survival for early stage-endometrial cancer. Obstet Gynecol.

[23] Kidd EA, Siegel BA, Dehdashti F, Rader JS, Mutic S, Mutch DG (2010). Clinical outcomes of definitive intensity-modulated radiation therapy with fluorodeoxyglucose–positron emission tomography simulation in patients with locally advanced cervical cancer. Int J Radiat Oncol Biol Phys.

